# Identification of critical base pairs required for CTCF binding in motif M1 and M2

**DOI:** 10.1007/s13238-017-0387-5

**Published:** 2017-03-17

**Authors:** Wufeng Li, Liping Shang, Kaimeng Huang, Jiao Li, Zhibin Wang, Hongjie Yao

**Affiliations:** 1 CAS Key Laboratory of Regenerative Biology, Joint School of Life Sciences, Guangzhou Medical University and Guangzhou Institutes of Biomedicine and Health, Chinese Academy of Sciences, Guangzhou, 510530 China; 2 College of Life Science, Shanxi Agricultural University, Taigu, 030801 China; 3 CAS Center for Excellence in Molecular Cell Science, Guangzhou Institutes of Biomedicine and Health, Chinese Academy of Sciences, Guangzhou, 510530 China; 4 Laboratory of Human Environmental Epigenome, Department of Environmental Health and Engineering, Bloomberg School of Public Health, Johns Hopkins University, Baltimore, MD 21205 USA

**Dear Editor,**

The ubiquitously expressed CCCTC-binding factor (CTCF), is highly conserved from *Drosophila* to mammals and plays multiple functions in the genome (Ohlsson et al., 2001). CTCF has been shown to establish chromatin insulation in vertebrate, and it also plays the roles in transcriptional regulation, X-chromosome inactivation, and imprinting of genes (Phillips and Corces, 2009). In addition, CTCF plays a pivotal role in genomic organization and loop formation by mediating long-range chromatin interactions between distant loci (Yao et al., 2010; Tang et al., 2015). Several hypotheses have been proposed to explain the diverse functions of CTCF. The popular ‘zinc-finger model’ proposed that the CTCF’s different functions are due to the interplay between the zinc-finger engagement and the underlying sequence differences (Ohlsson et al., 2001). Genome-wide studies have identified that the majority of CTCF binding sites belongs to a set of nonpalindromic CTCF binding sites with a consensus sequence referred to as M1 (Kim et al., 2007; Schmidt et al., 2012). Recently, another binding motif, referred to as M2 and 5–6 bp upstream of M1, has been discovered (Schmidt et al., 2012). Moreover, CTCF zinc fingers (ZFs) 4–8 strongly bind to the M1, while ZFs 7–11 tend to strongly bind to the M2 (Renda et al., 2007; Xiao et al., 2015). In this study, we aim to compare the binding abilities of CTCF to M1 and M2 and determine which bases were requirement for M1 and M2 bind to CTCF.

To investigate the binding capacities of CTCF-ZFs 1–11 to M1 or M2, pGEX-4T-2-CTCF-ZFs plasmid was constructed to induce the prokaryotic expression of GST-CTCF-ZFs (Fig. S1A). We tested three traditional temperature conditions (16°C, 28°C, and 37°C) and four IPTG concentrations (0.1 mmol/L, 0.5 mmol/L, 1 mmol/L, and 1.5 mmol/L). Coomassie blue staining results showed that GST-CTCF-ZFs was robustly induced at 28°C with an obvious band at about 55 kDa (Fig. S1B). We further optimized the IPTG concentration and found that the most suitable IPTG concentration for inducing GST-CTCF-ZFs expression was 0.6–0.7 mmol/L (Fig. S1C). Therefore, we concluded that the optimized induction condition for GST-CTCF-ZFs was 28°C with IPTG at a concentration between 0.6 and 0.7 mmol/L. GST-CTCF-ZFs was purified by using glutathione resin, eluted by using reduced glutathione and stained with reduced glutathione (Fig. S1D) and used for subsequent electrophoretic mobility shift assay (EMSA) experiments.

Next we synthesized two DNA oligos, containing the CTCF binding sites M1 and M2 (Fig. 1A and 1D). Our EMSA results indicated that 20 fmol biotin-labeled M1 could bind to 0.2 μg *in vitro* purified GST-CTCF-ZFs and lead to a supershift band (Fig. 1B). The binding of M1 to GST-CTCF-ZFs became stronger with the increased amount of GST-CTCF-ZFs from 0.2 μg to 2.5 μg (Fig. 1B). When the amount of GST-CTCF-ZFs increases to 1.5 μg, free DNA duplex was barely observed. To further examine the binding specificity, we performed EMSA with the competition of excessive amounts of unlabeled M1 DNA oligos. Our results demonstrated that the binding of purified GST-CTCF-ZFs to the biotin-labeled M1 oligo was competed by an excess amount of the unlabeled M1 oligo (Fig. 1C, lane 4).

**Figure 1:**
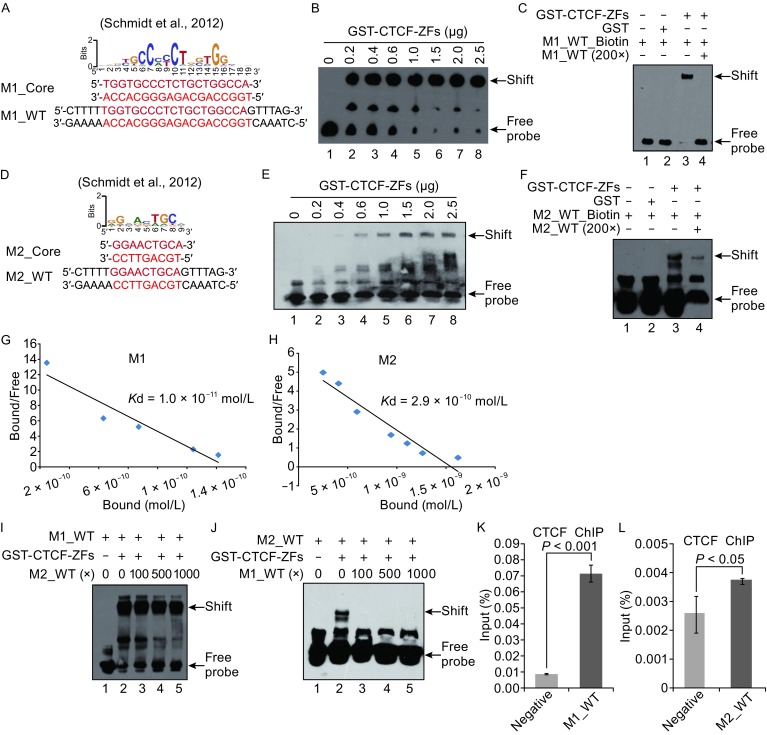
**CTCF-ZFs bind to M1 stronger than to M2**. (A) The sequences of CTCF binding motif M1. (B) Gel mobility shift analyses between M1 motif and increased amount GST-CTCF-ZFs fusion protein. In a volume of 20 μL, 20 fmol duplex probe was incubated with GST-CTCF-ZFs fusion protein with different amount (0, 0.2, 0.4, 0.6, 1.0, 1.5, 2.0, 2.5 μg). (C) Gel mobility shift analyses of competition of GST-CTCF-ZFs binding to the biotin-labeled M1 by an excess amount of unlabeled M1. Lane 1. No protein; Lane 2. Incubation of GST with biotin-labeled M1 probe; Lane 3. Incubation of GST-CTCF-ZFs with biotin-labeled M1 probe; Lane 4. Incubation of GST-CTCF-ZFs with 20 fmol biotin-labeled M1 and 4 pmol unlabeled M1 probe. (D) The sequences of CTCF binding motif 2. (E) Gel mobility shift analysis of GST-CTCF-ZFs protein with M2 motif. Assay condition was the same as in Fig. 1B. (F) Gel mobility shift analysis of competition of GST-CTCF-ZFs binding to the biotin-labeled M2 by an excess amount of unlabeled M2. Assay condition was the same as in Fig. 1B. (G) Scatchard analysis of the gel shift binding of M1 to GST-CTCF-ZFs. The ratio of bound to (not clear) free DNA is plotted versus the molar concentration of bound M1 in the reaction mixture. (H) Scatchard analysis of the gel shift binding of M2 to GST-CTCF-ZFs. The ratio of bound to free DNA is plotted versus the molar concentration of bound M2 in the reaction mixture. (I) Competition assays of CTCF-ZFs binding to biotin-labeled M1 oligo with different amount of unlabeled M2 oligo. (J) Competition assays of CTCF-ZFs binding to biotin-labeled M2 oligo with different amount of unlabeled M1 oligo. (K) The binding level of CTCF protein to CTCF binding motif 1 (M1) was quantitatively measured by qPCR using the indicated primer sets. M1 enrichment was represented as percentage of input (%). (L) The binding level of CTCF to CTCF binding motif 2 (M2) was quantitatively measured by ChIP-qPCR using the indicated primer sets

Compared with that of M1, the binding of M2 to purified GST-CTCF-ZFs protein was much weaker (Fig. 1E). No protein/DNA supershift was observed when the amount of GST-CTCF-ZFs in protein/DNA complex is between 0.2–0.4 μg (Fig. 1E, lanes 2 and 3). The supershift band gradually became stronger with the increased amount of GST-CTCF-ZFs from 0.6 μg to 2.5 μg (Fig. 1E). We further confirmed that the interaction between GST-CTCF-ZFs and the biotin-labeled M2 oligo was abolished by an excess amount of the unlabeled M2 oligos (Fig. 1F).

To quantify the strength of CTCF-ZF’s interaction with M1 and M2, we performed EMSA assays to determine the dissociation constant (*K*d) for CTCF-DNA interactions. The strong CTCF binding motif, M1 demonstrated a *K*d of 1.0 × 10^−11^ mol/L contrasting with *K*d values of 2.9 × 10^−10^ mol/L for CTCF-M2 interactions (Fig. 1G and 1H). Based on the amount of GST-CTCF-ZFs used in the EMSA assay and *K*d values, we hypothesized that, compared with the binding of M1 to GST-CTCF-ZFs, the binding of M2 to GST-CTCF-ZFs was much weaker (Fig. 1B–H). To test this hypothesis, we did two competition experiments that use unlabeled M1 or M2 to compete biotin-labeled M2 or M1. Our data indicated that the unlabeled M1 specifically and nearly completely displaced M2 binding at 100-fold excess, whereas an unlabeled M2 competitor oligo did not displace M1 binding even at 1000-fold excess (Fig. 1I and 1J).

To confirm our *in vitro* EMSA, we constructed two different plasmids with insertion of either M1 or M2 CTCF binding sites (Fig. S2). We transfected these constructs into 293T cells, respectively, and performed *in vivo* chromatin immunoprecipiation (ChIP). ChIP DNA was then examined by quantitative real-time PCR (ChIP-qPCR) experiments. We designed the qPCR primers in the construct at the regions with CTCF binding site (either M1 or M2) inserted and without CTCF binding site (Fig. S2). Our ChIP-qPCR results showed that the CTCF was recruited to both M1 and M2, respectively, but not to the negative region. Furthermore, stronger binding signals were observed at M1 in contrast to M2 (Fig. 1K and 1L), suggesting that CTCF prefers to bind to M1 rather than M2.

To determine the critical residues in CTCF binding site that are required for both M1 and M2 to bind to CTCF, we have looked into CTCF binding motif M1 and M2 and designed multiple point mutations according to the position weight matrix score (Schmidt et al., 2012). For this purpose, we synthesized M1 with a series of 3 bp mutations (Fig. 2A). We performed gel shift assays to compare the binding affinity of GST-CTCF-ZFs to the wild type and various mutated M1 (M1-Mut1 to M1-Mut7). Data suggested that, replacement of the “TGG” with “GTT” in M1 resulted in a drastic loss of the DNA binding ability of GST-CTCF-ZFs (Fig. 2B, lane 2), whereas other replacements (Mut2 to Mut7) did not alter binding signals significantly to that of wild-type M1 (Fig. 2B). These unexpected results indicate that “TGG” are most critical residues for M1 binding to CTCF. We also mutated one nucleotide or a few nucleotides within or around the “TGG”. However, these mutations did not abolish the binding shift of CTCF to M1 (Fig. 2C–F), except M1-Mut1. These data suggested that “TGG” might contribute the binding function of M1 more significantly.

**Figure 2:**
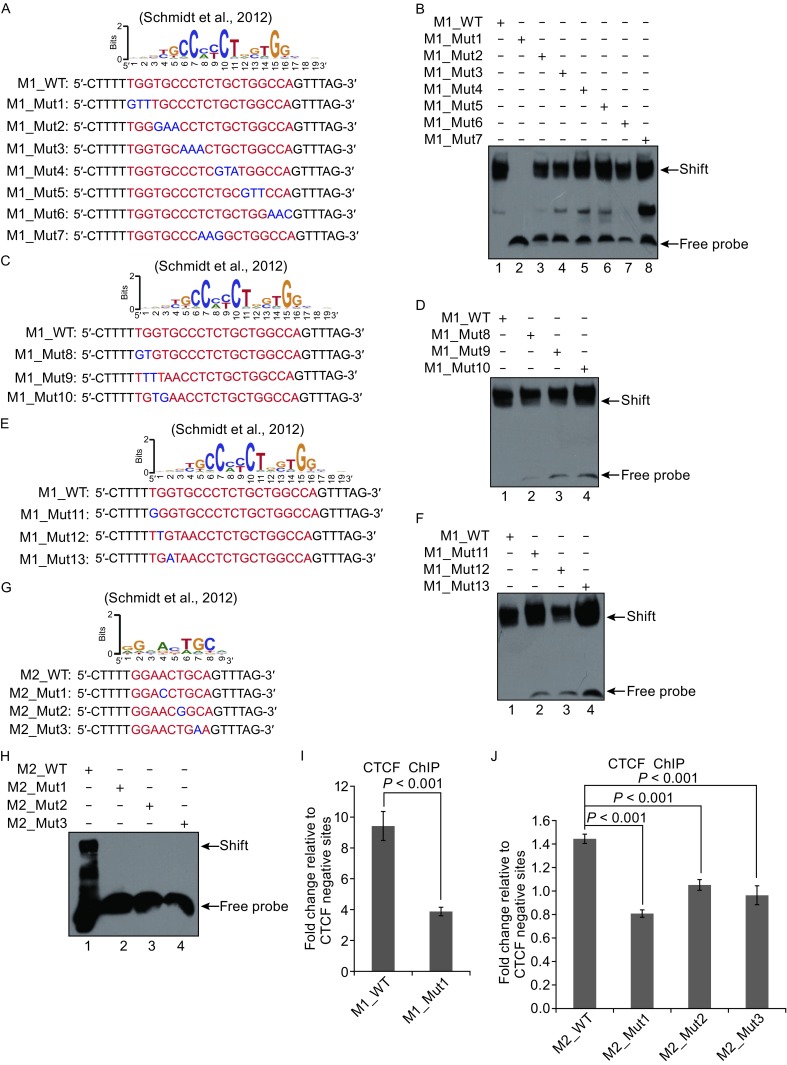
**Alteration of single/few nucleotide(s) in M1 or M2 dramatically impact(s) the binding of CTCF**. (A) The wild-type (WT) and 3 bp mutant (Mut) sequences of CTCF binding M1. (B) Comparisons of the binding capacity of WT M1 and mutant M1. The binding of GST-CTCF-ZFs fusion protein to WT M1 (lane 1), to Mut 1 (from TGG to GTT) (lane 2), to Mut 2 (from TGC to TAA) (lane 3), to Mut 3 (from CCT to AAA) (lane 4), to Mut 4 (from TGC to GTA) (lane 5), to Mut 5 (from TGG to GTT) (lane 6), to Mut 6 (from CCA to AAC) (lane 7), and to Mut 7 (from TCT to AAG) (lane 8). (C) The WT and 2 bp Mut sequences of CTCF binding M1. (D) Comparisons of the binding capacity of WT M1 and 2 bp Mut M1. The binding of GST-CTCF-ZFs fusion protein to WT M1 (lane 1), to Mut 8 (from TG to GT) (lane 2), to Mut 9 (from GG to TT) (lane 3), and to Mut 10 (from GT to TG) (lane 4). (E) The WT and 1 bp Mut sequences of CTCF binding M1. (F) Comparisons of the binding capacity of WT M1 and 1 bp Mut M1. The binding of GST-CTCF-ZFs fusion protein to WT M1 (lane 1), to Mut 11 (from T to G) (lane 2), to Mut 12 (from G to T) (lane 3), and to Mut 13 (from G to A) (lane 4). (G) The WT and 1 bp Mut sequences of CTCF binding M2. (H) Comparisons of the binding capacity of WT M2 and 1 bp Mut M2. The binding of GST-CTCF-ZFs fusion protein to WT M2 (lane 1), to Mut 1 (from A to T) (lane 2), to Mut 2 (from T to G) (lane 3), and to Mut 3 (from C to A) (lane 4). (I) Comparisons of the binding capacity of CTCF to both WT M1 and Mut M1 by ChIP-qPCR. (J) Comparisons of the binding capacity of CTCF to both WT M2 and Mut M2 by ChIP-qPCR

While CTCF contains 11 zinc fingers domains, the specificity and affinity can be controlled by a few crucial fingers (Renda et al., 2007). To further determine which zinc finger arrays actually bind to “TGG”, we used a web server (http://zf.princeton.edu/) that can predict DNA-binding specificities for C2H2-ZF-containing proteins, including CTCF (Persikov and Singh, 2014). Our analyses suggest that CTCF-ZFs 7–8 is critical for the M1 “TGG” binding (Sequence logos for the generated are given in Fig. S3).

To assess the position or critical residue that is important for M2 binding to CTCF, we made three point mutations (M2-Mut1, M2-Mut2, and M2-Mut3) according to position weight matrix score and performed EMSA assays (Fig. 2G). Our data showed that mutation of any selected single base within M2 abolished the binding of GST-CTCF-ZFs to M2 (Fig. 2H). These results suggested that the selected single base is required for the high-affinity interaction of M2 with CTCF.

To further verify that the mutated M1 or M2 abolished the CTCF binding of *in vivo*, we made several mutated constructs within CTCF binding sites (Fig. S2). *In vivo* ChIP experiments indicated that the mutation of M1 (from “TGG” to “GTT”) significantly decreased the binding of CTCF to M1 (Fig. 2I) and all single mutations within M2 abolished the binding of CTCF to M2 when compared to the control (Fig. 2J).

Several studies have reported that the transcription factor binding site sequence could play a role in fine-tuning the expression level of genes (Kandoth et al., 2013). For example, binding sites might be able to modulate gene expression as a consequence of differences in affinity (Bain et al., 2012), where high affinity binding sites induce a higher level of transcriptional activation than low affinity binding sites. In this respect, affinity of different CTCF binding motifs to CTCF-ZFs has not been determined. Herein we show that the binding abilities of GST-CTCF-ZFs to its M1 and M2 motifs were different. The binding of GST-CTCF-ZFs to M1 is much stronger than to M2. Importantly, similar conclusion was obtained with ChIP experiments (Fig. 1K and 1L).

When we initially incubated the GST-CTCF-ZFs with the oligos from either M1 or M2 core motif, we failed to see a clear shift after EMSA assay (Data not shown). Lobanenkov et al. suggested that additional DNA flanking outside the CTCF recognition motifs are required for tight binding but the exact sequence requirement for this flanking DNA may not be as strict as that of the CCCTC motifs (Lobanenkov et al., 1990). In combination with our results, we expect that the binding of CTCF to M1 and M2 *in vitro* need not only the core recognition sequence but also a few bps outside DNA. By referring to CTCF binding motif probes that detected by ChIP-seq (Xiao et al., 2015), we synthesized 30 bp biotin labeled double-strand M1 (5′-CTTTTTGGTGCCCTCTGCTGGCCAGTTTAG-3′) and 20 bp biotin labeled double-strand M2 (5′-CTTTTGGAACTGCAGTTTAG-3′) that including the core motif and additional flanking DNA sequence (5′-CTTTT and GTTTAG-3′) and tested their binding abilities to CTCF-ZFs in both *in vitro* and *in vivo* assays.

CTCF represses cancer cell growth and clonogenicity and has been classified as a candidate tumor suppressor gene (Rasko et al., 2001). Recent studies have identified mutations of human CTCF binding sites in various human cancer types including Wilms’ tumor, leukaemia (Mullighan et al., 2011). Thus, mutation of CTCF binding sites at specific loci may dysregulate the expressions of tumor suppression genes or oncogenes, thereby contributing to the malignant phenotype (Filippova et al., 2002). In this study, we found that several mutations of CTCF binding sites abolished the binding of CTCF-ZFs to the mutated sites both *in vitro* and *in vivo*. These mutations are likely to exist in the genome of some cancer types. To enrich our knowledge for the roles of all base pairs in CTCF binding sites, a genetic mutation screening might be necessary for us to implement the mutations in a variety of patterns in the future.

## Electronic supplementary material

Below is the link to the electronic supplementary material.

Supplementary material 1 (PDF 162 kb)

Supplementary material 2 (PDF 1699 kb)

Supplementary material 3 (PDF 14 kb)
