# Supplementary material for: Identification of critical base pairs required for CTCF binding in motif M1 and M2
Source: Protein Cell. 2017 Mar 17;8(7):544–9. doi: 10.1007/s13238-017-0387-5 (PMC5498337; doi:10.1007/s13238-017-0387-5)
Supplement: Supplementary file 1 — Supplementary material 1 (PDF 162 kb) [file 13238_2017_387_MOESM1_ESM.pdf]

Materials and Methods

Cell Culture and Transfection

293T cells were cultured in DMEM (Hyclone) supplemented with 10% fetal bovine serum. Cells were maintained at 37 ℃ in a 5% CO_2 incubator. After reaching 70% confluence, 293T cells were transfected with 10 μg pFlag-CMV-4 plasmids using PEI.

Plasmid Constructions

pGEX-4T-2-CTCF-ZFs expression vector was constructed as below. DNA fragments encoding the human CTCF-ZFs were generated by PCR using the plasmid pOZN-hCTCF as a DNA template. The primers for PCR are shown in Table S1. PCR products were digested with the restriction enzymes *XhoI* I and *Sal* I and cloned into pGEX-4T-2 bacterial expression vector. The plasmids were sequenced to confirm that there were no point mutations in the coding sequences.

pFlag-CMV-4 vector containing M1 or M2 was constructed by using the following strategy: M1/M2 and mutation probes were inserted into pFlag-CMV-4 vector by digesting with both *Eco*R I and *Xba* I. The sequences of M1/M2 and mutated probes were shown in Table S3. Both forward and reverse oligos were annealed into double oligos, which were double digested by *Eco*R I and *Xba* I. The resulting fragments were cloned into pFlag-CMV-4 vector. The correct constructs were confirmed by sequencing.

Induction and Purification of the Proteins

The fusion proteins were expressed in the *E. coli* BL21 host strain as previously described (Renda et al., 2007)(Yao et al., 2010). The transformed cells were grown in LB with 100 mg/mL of ampicillin at 37 ℃ until the absorbance at 600 nm was 0.6-0.8, at which time the medium was supplemented with 200 μM $ZnSO_4$. The protein expression was induced with 0.7 mM Isopropyl β-D-1-thiogalactopyranoside (IPTG) at 28 ℃ for 6 hr. Then the cells were harvested and resuspended in 1 × PBS (pH 7.4) containing the protease inhibitors (1 mM PMSF, 1 mM Protease inhibitor cocktail). After that, the cells were sonicated and supernatant fraction was collected by centrifuging at 4000 × rpm for 15 min. The supernatant was then filtered with 0.45 μm membrane, then was incubated with Glutathione Resin (Gene Script, L00206) for 4 hr. After washing with 1% PBS-T, the purified fractions were eluted with glutathione elution buffer (10 mM glutathione, 100 mM Tris (pH8.0), and 100 mM NaCl).

Electrophoretic Mobility Shift Assay (EMSA)

DNA probe sequences were listed in Table S2. M1 and M2 were synthesized and biotin-labeled at 3'-end from IGE company. The mutated probes were labeled by using the EMSA Probe Biotin 3'-end Labeling Kit (Beyotime, GS008). EMSA was performed using Light Shift Chemiluminescent EMSA reagents as described in the manufacturer’s manual (Thermo, 20148). Briefly, the probes were incubated with purified proteins in 1 × binding buffer at room temperature for 20 min. The binding complex was electrophoresed on 5% nondenaturing polyacrylamide gels in ice-cold

0.5 × TBE buffer (45 mM Tris-borate, 1 mM EDTA, pH 8.0). The gel was electrotransferred to a nylon membrane in ice-cold 0.5 × TBE buffer. After crosslinking using UV-Linker for 10 min, the membrane was incubated with stabilized streptavidin-horseradish peroxidase conjugate and rinsed with the washing buffer. The biotin-labeled DNA was then detected by chemiluminescence using the Chemiluminescent Nucleic Acid Detection Module (Thermo, 89880). Detected bands were measured and analyzed by computer quantification using the Quantity One Manual Software.

Chromatin Immunoprecipitation and Quantitative Real-time PCR (ChIP-qPCR)

ChIP-qPCR assays were performed as previously described (Huang et al., 2013). Briefly, about 1×10^7 293T cells were cross-linked with 1% formaldehyde at room temperature for 10 min. Then the reaction was stopped by adding glycine (final concentration, 0.125 M) for 5 min. The cells were sonicated in SDS lysis buffer to achieve a chromatin size of 100-500 bp. The sonicated chromatin was diluted by using ChIP dilution buffer (0.01% SDS, 1.1% Triton-100, 1.2 mM EDTA, 16.7 mM Tris-Cl (pH 8.0), and 167 mM NaCl). About 1 μg of anti-CTCF antibody (Millipore, #07-729) was coupled with Dynabead protein A and G (1:1 mixed). Then, the mixture was incubated with chromatin lysates overnight at 4℃ with rotation. Immune complexes were washed with the following buffers: low-salt wash buffer (0.1% SDS, 1% Triton X-100, 2 mM EDTA, 20 mM Tris-Cl (pH8.0) and 150 mM NaCl), high-salt wash buffer (0.1% SDS, 1% Triton X-100, 2 mM EDTA, 20 mM Tris-Cl (pH 8.0) and

500 mM NaCl), LiCl wash buffer (0.25 M LiCl, 1% IGEPAL-CA630, 1% deoxycholic acid (sodium salt), 1 mM EDTA, and 10 mM Tris-Cl (pH8.0)) and TE buffer (10 mM Tris-Cl (pH8.0) and 1 mM EDTA). Antibody-bound chromatin was reverse-cross-linked, and the ChIP DNA samples were purified for PCR reaction. Primers used for ChIP-qPCR were listed in Table S4. Each PCR reaction was performed at least three times.

Reference

Huang, K., Jia, J., Wu, C., Yao, M., Li, M., Jin, J., Jiang, C., Cai, Y., Pei, D., Pan, G., *et al.* (2013). Ribosomal RNA gene transcription mediated by the master genome regulator protein CCCTC-binding factor (CTCF) is negatively regulated by the condensin complex. J Biol Chem 288, 26067-26077.

Renda, M., Baglivo, I., Burgess-Beusse, B., Esposito, S., Fattorusso, R., Felsenfeld, G., and Pedone, P.V. (2007). Critical DNA binding interactions of the insulator protein CTCF: a small number of zinc fingers mediate strong binding, and a single finger-DNA interaction controls binding at imprinted loci. J Biol Chem 282, 33336-33345.

Yao, H., Brick, K., Evrard, Y., Xiao, T., Camerini-Otero, R.D., and Felsenfeld, G. (2010). Mediation of CTCF transcriptional insulation by DEAD-box RNA-binding protein p68 and steroid receptor RNA activator SRA. Genes Dev 24, 2543-2555.

Supplementary Figure Legends

Figure S1. Expression and purification of GST-CTCF-ZFs recombinant protein.

A. Agarose gel examination of PCR product for CTCF-ZFs and pGEX-4T-2-CTCF-ZFs: Lane 1. Marker; Lane 2. PCR product amplified for CTCF-ZFs by using specific primers; Lane 3. DNA fragments after digesting pGEX-4T-2 plasmid with Xhol I and Sal I; Lane 4. Undigested pGEX-4T-2-CTCF-ZFs plasmid; Lane 5. DNA fragments after digesting pGEX-4T-2-CTCF-ZFs with Xhol I and Sal I.

B. Effect of different temperatures on the induction of GST-CTCF-ZFs fusion protein. Lane 1. Marker; Lane 2. Coomassie blue staining for the protein from uninduced pGEX-4T-2-CTCF-ZFs *BL21* stain; Lane 3-6. Coomassie blue staining for the proteins induced by IPTG from pGEX-4T-2-CTCF-ZFs *BL21* stain at 16 ℃; Lane 7-10. Coomassie blue staining for the proteins induced by IPTG from pGEX-4T-2-CTCF-ZFs *BL21* stain at 28 ℃; Lane 11-14. Coomassie blue staining for the proteins induced by IPTG from pGEX-4T-2-CTCF-ZFs *BL21* stain at 37 ℃.

C. Effect of IPTG concentrations on the induction of GST-CTCF-ZFs fusion protein. Lane 1. Marker; Lane 2-10. IPTG with different concentrations from 0.5-1.2 mM.

D. Purification of GST-CTCF-ZFs recombinant protein. Lane 1. Marker; Lane 2. Supernatant of the sonicated and filtered bacteria lysate; Lane 3. Examination of GST-CTCF-ZFs recombinant protein after GST purification; Lane 4.

GST-CTCF-ZFs fusion protein after eluting with the reduced glutathione elution buffer.

Figure S2. The schematic diagram summarizing the ChIP-qPCR assay.

The CTCF binding sequences (WT/Mut) were inserted into pFlag-CMV-4 plasmid.

Figure S3. Predicted sequence logos of CTCF-ZF binding sites.

A-K. sequence logos of CTCF-ZF 1 to CTCF-ZF 11 binding sites predicted by SVM.
