# Supplementary material for: Identification of critical base pairs required for CTCF binding in motif M1 and M2
Source: Protein Cell. 2017 Mar 17;8(7):544–9. doi: 10.1007/s13238-017-0387-5 (PMC5498337; doi:10.1007/s13238-017-0387-5)
Supplement: Supplementary file 2 — Supplementary material 2 (PDF 1699 kb) [file 13238_2017_387_MOESM2_ESM.pdf]

Figure S1

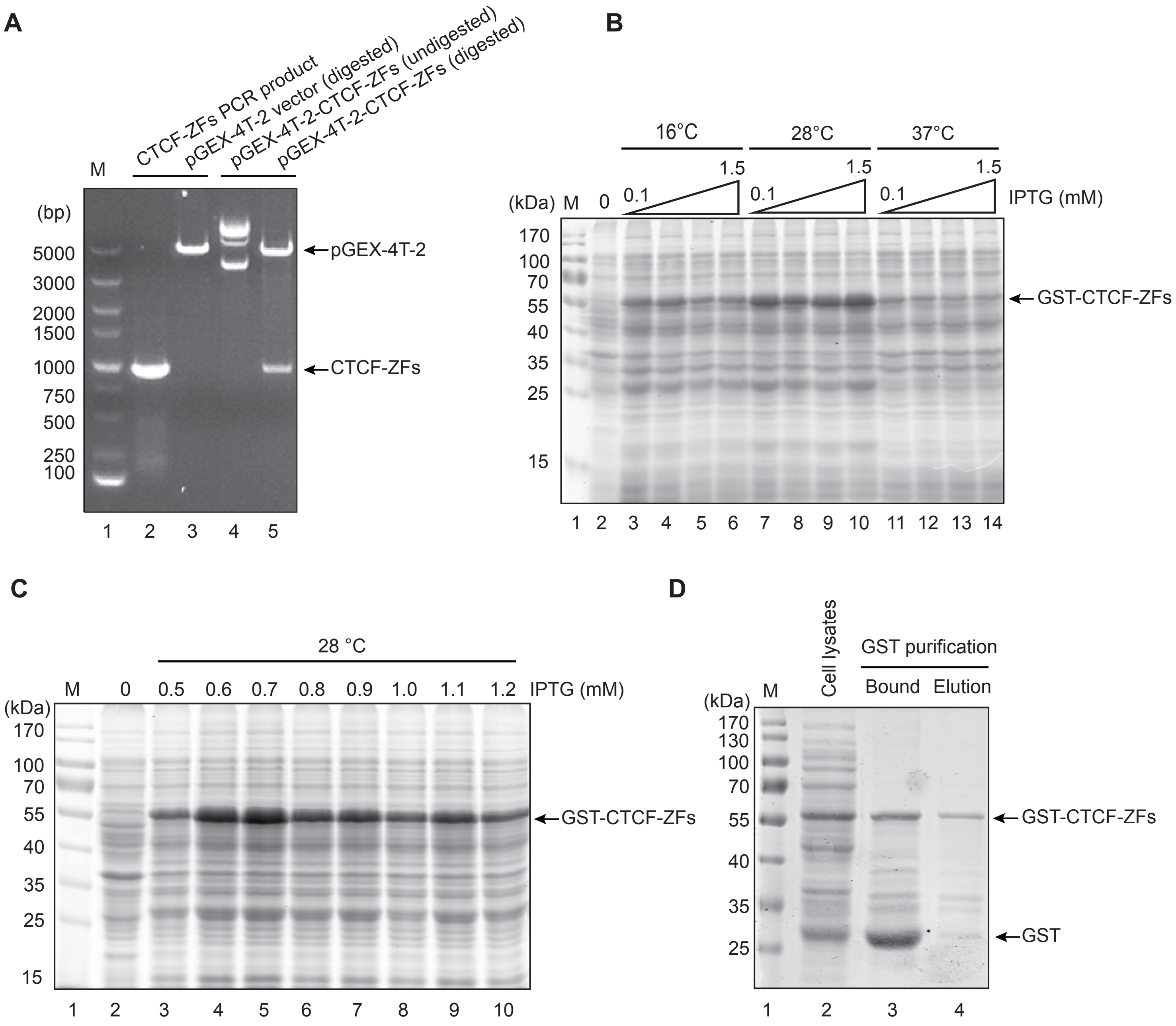

Figure S1. Expression and purification of GST-CTCF-ZFs recombinant protein.

A. Agarose gel examination of PCR product for CTCF-ZFs and pGEX-4T-2-CTCF-ZFs: Lane 1. Marker; Lane 2. PCR product amplified for CTCF-ZFs by using specific primers; Lane 3. DNA fragments after digesting pGEX-4T-2 plasmid with XhoI I and Sal I; Lane 4. Undigested pGEX-4T-2-CTCF-ZFs plasmid; Lane 5. DNA fragments after digesting pGEX-4T-2-CTCF-ZFs with XhoI I and Sal I.

B. Effect of different temperatures on the induction of GST-CTCF-ZFs fusion protein. Lane 1. Marker; Lane 2. Coomassie blue staining for the protein from uninduced pGEX-4T-2-CTCF-ZFs *BL21* stain; Lane 3-6. Coomassie blue staining for the proteins induced by IPTG from pGEX-4T-2-CTCF-ZFs *BL21* stain at 16°C; Lane 7-10. Coomassie blue staining for the proteins induced by IPTG from pGEX-4T-2-CTCF-ZFs *BL21* stain at 28°C; Lane 11-14. Coomassie blue staining for the proteins induced by IPTG from pGEX-4T-2-CTCF-ZFs *BL21* stain at 37°C.

C. Effect of IPTG concentrations on the induction of GST-CTCF-ZFs fusion protein. Lane 1. Marker; Lane 2-10. IPTG with different concentrations from 0.5-1.2 mM.

D. Purification of GST-CTCF-ZFs recombinant protein. Lane 1. Marker; Lane 2. Supernatant of the sonicated and filtered bacteria lysate; Lane 3. Examination of GST-CTCF-ZFs recombinant protein after GST purification; Lane 4. GST-CTCF-ZFs fusion protein after eluting with the reduced glutathione elution buffer.

Figure S2

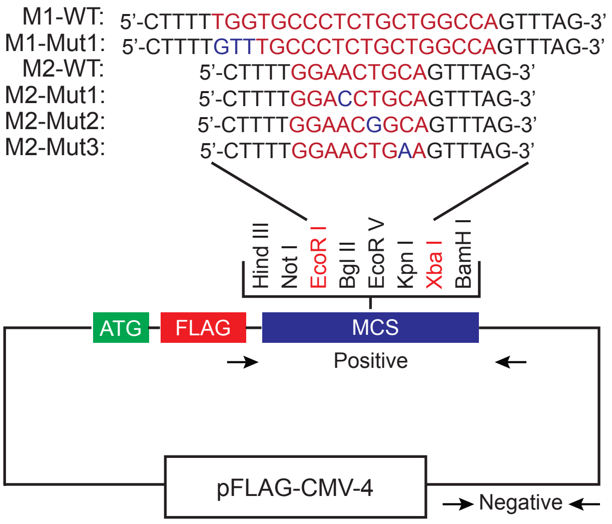

Figure S2. The schematic diagram summarizing the ChIP-qPCR assay.

The CTCF binding sequences (WT/Mut) were inserted into pFlag-CMV-4 plasmid.

Figure S3

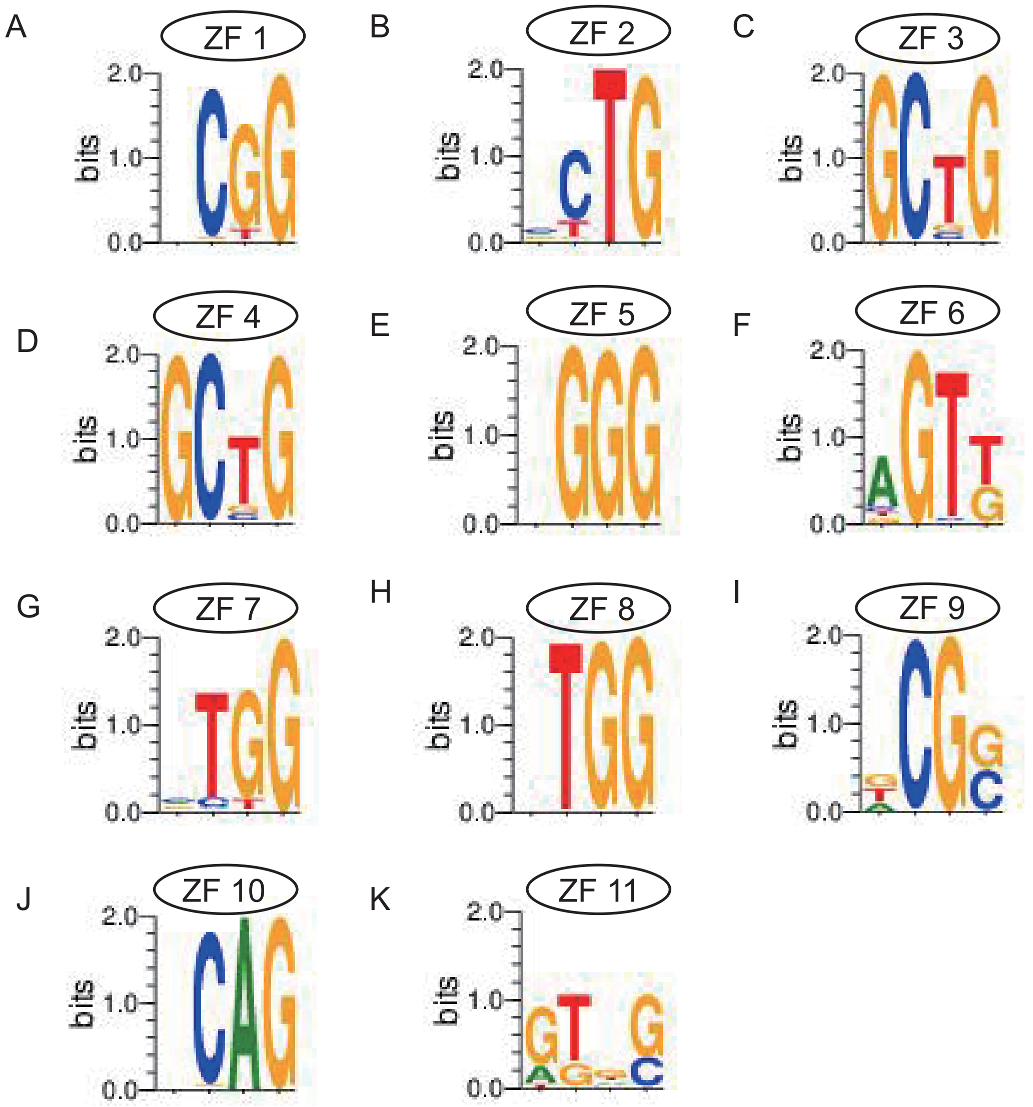

Figure S3. Predicted sequence logos of CTCF-ZF binding sites.

A-K. Sequence logos of CTCF-ZF 1 to CTCF-ZF 11 binding sequences predicted by SVM.
