# Supplementary material for: Identification of critical base pairs required for CTCF binding in motif M1 and M2
Source: Protein Cell. 2017 Mar 17;8(7):544–9. doi: 10.1007/s13238-017-0387-5 (PMC5498337; doi:10.1007/s13238-017-0387-5)
Supplement: Supplementary file 3 — Supplementary material 3 (PDF 14 kb) [file 13238_2017_387_MOESM3_ESM.pdf]

Supplementary Table S1. Sequence of primers used for constructing pGEX-4T-2-hCTCF-ZF expression vector.

**Name**	**Sequence (from 5' to 3')**
pGEX-4T-2-hCTCF-ZF-F	5'-CCGCTCGAGCGGTGTAAAGAAGACATTCCAGTG-3'
pGEX-4T-2-hCTCF-ZF-R	5'-ACGCGTCGACTCAGCCATCTGGGCCAGCACAATTATC-3'

Supplementary Table S2. Sequence of probes used for EMSA experiments.

**Name**	**Sequence (from 5' to 3')**
M1-F	5'-CTTTTTGGTGCCCTCTGCTGGCCAGTTTAG-3'
M1-R	5'-CTAAACTGGCCAGCAGAGGGCACCAAAAAG-3'
M2-F	5'-CTTTTGGAACTGCAGTTTAG-3'
M2-R	5'-CTAAACTGCAGTTCCAAAAG-3'
M1-Mut1-F	5'-CTTTTGTTTGCCCTCTGCTGGCCAGTTTAG-3'
M1-Mut1-R	5'-CTAAACTGGCCAGCAGAGGGCAAACAAAAG-3'
M1-Mut2-F	5'-CTTTTTGGGAACCTCTGCTGGCCAGTTTAG-3'
M1-Mut2-R	5'-CTAAACTGGCCAGCAGAGGTTCCCAAAAAG-3'
M1-Mut3-F	5'-CTTTTTGGTGCAAACTGCTGGCCAGTTTAG-3'
M1-Mut3-R	5'-CTAAACTGGCCAGCAGTTTGCACCAAAAAG-3'
M1-Mut4-F	5'-CTTTTTGGTGCCCTCGTATGGCCAGTTTAG-3'
M1-Mut4-R	5'-CTAAACTGGCCATACGAGGGCACCAAAAAG-3'
M1-Mut5-F	5'-CTTTTTGGTGCCCTCTGCGTTCCAGTTTAG-3'
M1-Mut5-R	5'-CTAAACTGGAACGCAGAGGGCACCAAAAAG-3'
M1-Mut6-F	5'-CTTTTTGGTGCCCTCTGCTGGAACGTTTAG-3'
M1-Mut6-R	5'-CTAAACGTTCCAGCAGAGGGCACCAAAAAG-3'
M1-Mut7-F	5'-CTTTTTGGTGCCCAAGGCTGGCCAGTTTAG-3'
M1-Mut7-R	5'-CTAAACTGGCCAGCCTTGGGCACCAAAAAG-3'
M1-Mut8-F	5'-CTTTTGGGTGCCCTCTGCTGGCCAGTTTAG-3'
M1-Mut8-R	5'-CTAAACTGGCCAGCAGAGGGCACCCAAAAG-3'
M1-Mut9-F	5'-CTTTTTTGTGCCCTCTGCTGGCCAGTTTAG-3'

M1-Mut9-R	5’-CTAAACTGGCCAGCAGAGGGCACAAAAAAG-3’
M1-Mut10-F	5’-CTTTTTGTTGCCCTCTGCTGGCCAGTTTAG-3’
M1-Mut10-R	5’-CTAAACTGGCCAGCAGAGGGCAACAAAAAG-3’
M1-Mut11-F	5’-CTTTTGTGTGCCCTCTGCTGGCCAGTTTAG-3’
M1-Mut11-R	5’-CTAAACTGGCCAGCAGAGGGCACACAAAAG-3’
M1-Mut12-F	5’-CTTTTTTTTGCCCTCTGCTGGCCAGTTTAG-3’
M1-Mut12-R	5’-CTAAACTGGCCAGCAGAGGGCAAAAAAAAG-3’
M1-Mut13-F	5’-CTTTTTGTGGCCCTCTGCTGGCCAGTTTAG-3’
M1-Mut13-R	5’-CTAAACTGGCCAGCAGAGGGCCACAAAAAG-3’
M2-Mut1-F	5’-CTTTTGGACCTGCAGTTTAG-3
M2-Mut1-R	5’-CTAAACTGCAGGTCCAAAAG-3
M2-Mut2-F	5’-CTTTTGGAACGGCAGTTTAG-3
M2-Mut2-R	5’-CTAAACTGCCGTTCCAAAAG-3
M2-Mut3-F	5’-CTTTTGGAACTGAAGTTTAG-3
M2-Mut3-R	5’-CTAAACTTCAGTTCCAAAAG-3

Supplementary Table S3. Sequence of oligos used for constructing pFlag-CMV-4 vector.

**Name**	**Sequence (from 5’ to 3’)**
M1-F	5’-AATTCTTTTTGGTGCCCTCTGCTGGCCAGTTTAG-3’
M1-R	5’-CTAGCTAAACTGGCCAGCAGAGGGCACCAAAAAG-3’
M2-F	5’-AATTCTTTTGGAACTGCAGTTTAG-3’
M2-R	5’-CTAGCTAAACTGCAGTTCCAAAAG-3’
M1-Mut1-F	5’-AATTCTTTTGTTTGCCCTCTGCTGGCCAGTTTAG-3’
M1-Mut1-R	5’-CTAGCTAAACTGGCCAGCAGAGGGCAAACAAAAG-3’
M2-Mut1-F	5’-AATTCTTTTGGACCTGCAGTTTAG-3
M2-Mut1-R	5’-CTAGCTAAACTGCAGGTCCAAAAG-3
M2-Mut2-F	5’-AATTCTTTTGGAACGGCAGTTTAG-3
M2-Mut2-R	5’-CTAGCTAAACTGCCGTTCCAAAAG-3

M2-Mut3-F	5’-AATTCTTTTGGAACTGAAGTTTAG-3
M2-Mut3-R	5’-CTAGCTAAACTTCAGTTCCAAAAG-3

Supplementary Table S4. Sequence of primers used for ChIP-qPCR.

**Name**	**Sequence (from 5’ to 3’)**
pFlag-negative-F	5’-GAGTGTGTCTTCCCGTTTTCCG-3’
pFlag-negative-R	5’-CCTATGGAAAAACGCCAGCAAC-3’
pFlag-positive-F	5’-ATGGACTACAAAGACGATGACG-3’
pFlag-positive-R	5’-ACTGGGGAGGGGTCACAGGGAT-3’
